# Multi-phase dataset for bulk Ti and the Ti-6Al-4V alloy

**DOI:** 10.1038/s41597-025-05302-3

**Published:** 2025-12-05

**Authors:** Connor S. Allen, Albert P. Bartók

**Affiliations:** 1 Department of Physics, University of Warwick, Coventry, CV4 7AL, United Kingdom; 2 Warwick Centre for Predictive Modelling, School of Engineering, University of Warwick, Coventry, CV4 7AL, United Kingdom

**Keywords:** Atomistic models, Structure of solids and liquids

## Abstract

Titanium and its alloys are technologically important materials that display a rich phase behaviour. In order to enable large-scale, realistic modelling of Ti and its alloys on the atomistic scale, MLIPs are crucial, but rely on databases of atomic configurations. We report databases of such configurations that represent the *α*, *β*, *ω* and liquid phases of Ti and the Ti-6Al-4V alloy, where we provide total energy, force and stress values evaluated by DFT using the PBE functional. We have also utilised a data reduction strategy, via non-diagonal supercells, for the vibrational properties of Ti and sampling of atomic species for Ti-6Al-4V. These configurations may be used to fit models that can accurately model the phase behaviour of Ti and Ti-6Al-4V across a broad range of thermodynamic conditions. To validate models, we assembled a set of benchmark protocols, which can be used to rapidly develop and evaluate MLIP models. We demonstrated the utility of our databases and validation tools by fitting models using the GAP and ACE frameworks.

## Background and summary

The transition metal Ti and its ternary alloy Ti-6Al-4V (Ti 90 wt%, Al 6 wt%, V 4 wt%) have industrial relevance in aerospace, biomedical, defence and high-performance engineering applications due to the machinability, anti-corrosive and high strength-to-weight properties of the material^1,2^. Pure Ti has been experimentally observed to form in the *α* (hexagonal close packed (hcp), P6_3_/mmc) phase at ambient conditions, undergoing a transformation to the *β* (body-centred cubic (bcc), Im-3m) at approximately 1150 K^3–5^ at ambient pressure. First-principles modelling shows the *ω*-Ti (hexagonal, P6/mmm) phase to be the ground state^4,6–12^. The relative stability of Ti at high pressure has also been investigated extensively by both computational^4,6–12^ and experimental^4,8,13–18^ studies, with significant controversy surrounding the phase transition boundary of *α* → *ω* and the existence of high pressure phases. Experimental studies have established that Ti-6Al-4V forms a polycrystalline solid at ambient conditions, predominantly of the *α* structure with the existence of interspersed *β* grains between phase boundaries, of which contain large concentrations of V and reduced Al content^19^. Similarly to pure Ti, the associated phase diagrams of Ti-6Al-4V reveal that the predominant solid phases of interest of this alloy include the *α*, *β* and *ω* phases^20,21^.

Machine Learning Interatomic Potentials (MLIPs) have recently emerged as surrogate models of the *ab initio* Born-Oppenheimer (PES) that retain first-principles accuracy at a very moderate computational cost and linear scaling with system size^22–27^. These models are built by carrying out non-linear regression to reproduce microscopic observables obtained from *ab initio* calculations, e.g. total energies, forces and stresses, as a function of the atomic positions. The mapping of atomic positions is usually encoded such that a given atomic environment is invariant under translations, rotations, and permutations of identical species, where these encoders are often called descriptors, symmetry functions or fingerprints. Examples include the Smooth Overlap of Atomic Positions (SOAP)^28^ and ACE^24^. These surrogate models can maintain *ab initio* accuracy whilst significantly reducing the computational cost associated with evaluating an atomic configuration, when compared against the underlying *ab initio* method used to develop the MLIP.

Our main result is that we constructed databases of atomic configurations, labelled by *ab initio* calculations, of Ti and Ti-6Al-4V representing multiple thermodynamically stable phases below 30 GPa for use in MLIP development. We have also developed a set of benchmarks that may be used to validate any MLIP fitted using our database. Finally, we utilised the (GAP) and (ACE) frameworks to fit MLIPs to demonstrate the coverage and sufficiency of our database.

## Methods

### Density Functional Theory

The *ab initio* calculations that provided the labels (total energies, forces and stresses) in the training database and reference benchmarks were performed using the plane-wave Density Functional Theory (DFT) code, CASTEP (v24.1)^29^. On-the-fly ultrasoft pseudopotentials were also generated for Al, V and Ti with respective valence electronic structures: 3s^2^ 3p^1^, 3s^2^3p^6^3d^2^4s^2^, and 3s^2^3p^6^3d^2^3s^2^. The PBE^30^ functional was used to approximate exchange-correlation. Parameters of DFT calculations are set such that our calculations are converged to sub-meV/atom relative to a computationally excessive basis. We have found that this level of convergence can be achieved by applying a plane-wave energy cut off of 800 eV, and sampling the electronic Brillouin Zone (BZ) using a Monkhorst-Pack grid that had a spacing of 0.02 Å^−1^. We have applied a smearing width of 0.05 eV on the electronic states to ensure reliable convergence of the calculations.

To obtain reference atomic configurations, geometry optimisations were performed for the experimentally observed symmetries of Ti below 12 GPa with a maximum force tolerance of less than 1 meV/Å a stress tolerance of less than 0.1 GPa, and energy tolerance of 10^−9^ eV/atom for Self-Consistent Field (SCF) cycles. This provided the relaxed lattice parameters for each crystalline phase, which was later used for generating training data and benchmark calculations. The geometry relaxations of the physically relevant phases in pure Ti were also used as the basis for constructing the Ti-6Al-4V dataset.

#### Training database

Representing the exact stoichiometry of the Ti-6Al-4V alloy would require the minor alloying components represent 10.2% Al and 3.6 % V by number. To approximate this stoichiometry within 2%, one may construct approximate primitive cells for each crystalline phase being considered. For the *α* and *β* phases the smallest such configuration, containing at least 1 V atom per unit cell, contains 28 atoms (3 × 2 × 2 and 4 × 3 × 2 respectively) with 3 Al and 1 V (10.7% Al and 3.6 % V by number), and for the *ω* phase the a 24 atom supercell (2 × 2 × 2) may be considered with 3 Al and 1 V (12.5% Al and 4.2 % V). The maximise of the coverage of the database, we considered a random substitutions of Ti by the other alloying elements. In order to save computational resources, we aimed to construct a dataset using a minimal number of atoms per DFT calculation in a given periodic cell, whilst sampling the possible disorder, both vibrational and substitutional, efficiently. This then motivated the Non-Diagonal Supercell (NDSC) approach^31,32^ to constructing crystalline configurations for *ab initio* database building, and this extended version of the NDSC strategy is outlined later.

#### Validation database

Validation of any MLIP and the database it is based on should be tailored to probe the exact quantity of interest, therefore we did not attempt to assemble a generic protocol for Ti or Ti-6Al-4Al. Our benchmarks presented here are intended to assess the broad validity and coverage of the training database to study phase stability, elastic and vibrational properties.

For validation, we created larger periodic unit cells of atomic configurations such that we can represent a more realistic disorder of the minor alloying components in a simulation cell. The size of these atomic structure models, for each crystalline phase, were chosen to be as large as tractably possible for calculation of DFT labels. These configurations are intentionally left out of the training data and we refer to them as the validation dataset when evaluating energies, forces and virial stresses of the developed MLIPs against our reference DFT.

We use these benchmark configurations to evaluate the NDSC strategy, which requires relatively small unit cells to constructing data points for medium-entropy crystalline systems. The performance of MLIP models fitted using the NDSC data can be validated against the DFT predictions using these atomic configurations.

Utilising the orthorhombic ground state geometries of pure Ti as a starting point, we first generated supercells for the *β* (4 × 4 × 4, 128 atoms), *α* (3 × 3 × 3, 108 atoms) and *ω* (2 × 3 × 3, 108 atoms) crystalline structures. From these atomic configurations, we also generate isotropic volume perturbations of 96% and 98% of the ground state volume per atom of pure Ti for crystalline system, providing data points to study transferability of MLIPs to high pressure. The atomic positions within the unit cells are then displaced from ideal lattice sites according to a normal distribution with standard deviation of 0.10 Å, such that each volume perturbation had 3 such samples. The atomic species were set randomly such that we recover the stoichiometry of Ti-6Al-4V in each configuration to within 1 %. In our benchmarks, we evaluate the surrogate MLIP on these configurations and compare against the DFT labels.

#### Elastic behaviour

In order to evaluate the response of the MLIPs to cell deformation, we compute the elastic constants for each crystalline symmetry with DFT as a benchmark. In these calculations we construct supercells containing 3 × 3 × 3 (*α*, *ω*) and 4 × 4 × 4 (*β*) repeating units of the primitive cell for each crystalline phase. In these configurations we substitute the alloying components using the special quasirandom structures^33^ algorithm within the integrated cluster expansion toolkit^34^ python library, on the approximate unit cells. The cell vectors and atomic positions of these structures were relaxed using DFT, and elastic constants were fit utilising the finite differences method implemented within the matscipy package^35^.

#### Vibrational properties

To calculate our reference phonon dispersions and density of states, firstly geometry optimisations were performed on each atomic structure until a structure with maximum force of less than 1 meV/Å is found, with the stress change tolerance below 0.1 GPa, with an energy tolerance of 10^−9^ eV used for SCF cycles. We calculated the force constant matrices utilising the finite displacement method within CASTEP, using a finite displacement of 0.02 Å (0.01 Å for pure Ti), in supercells corresponding to a uniform 4 × 4 × 4 grid in the vibrational BZ. We calculate the phonon dispersions along high symmetry lines for each crystalline symmetry^36^. Phonon density of states was calculated on a uniform 40 × 40 × 40 **q** grid of the vibrational BZ.

Calculating phonon dispersions requires the replication of a simulation cell so that one can accurately sample between high symmetry points in the vibrational BZ. In the case of pure Ti, this is easily tractable with DFT, as the primitive cell structures for each crystalline phase contain only a few atoms, however, utilising the approximate primitive unit cells of Ti-6Al-4V would have required excessive computational effort, as the unit cells need to be larger to accommodate the stoichiometry of the alloy. For this reason, when evaluating phonon dispersions relevant to Ti-6Al-4V, we instead calculate the phonon dispersions, density of states, and harmonic free energies (for dynamically stable structures) for a series of smaller supercells (no more than 12 atoms) in which for every crystal symmetry a Al-Al, Al-V and V-V interaction as nearest neighbours is being considered. Inevitably, these configurations contain a significantly higher ratios of the alloying elements, nevertheless, they provide valuable benchmark data against which MLIP models can be compared.

Interactions between Al and V atoms occupying nearby atomic sites have been found to be an energetically favourable^37^, and our previous results indicate that Al-Al ordering is likely disfavoured. We utilise these benchmark configurations specifically to assess the performance of surrogate MLIP models on capturing the interactions of minor alloying components, as similar configurations are more sparsely represented within in the training data.

### Database generation for Ti phases

As plane wave DFT scales excessively with the number of atoms simulated (${\mathcal{O}}({N}^{3})$), to capture each crystalline system effectively we utilised NDSCs^32^ as a basis for representing the vibrational properties and substitutional disorder. This strategy allows for a much more efficient sampling of the vibrational BZ, and as a result, requires less computational effort to achieve a given accuracy^31^ for a fitted MLIPs model. We considered NDSCs commensurate with the sampling achieved using 4 × 4 × 4 supercells of the primitive unit cells. This results in no more than 4 repeating units of the primitive unit cell for each crystal symmetry, efficiently limiting the total number of atoms required in the DFT calculations. For each DFT, volume perturbations were generated by isotropically straining the cell such that points along the pressure axis are uniformly sampled. The configurations then had the atomic coordinates randomly perturbed by a normal distribution with standard deviation of 0.10 Å. Additional data was generated for the *α* and *ω* phases that utilise the same volume perturbations, however, normally distributing atomic positions around ideal lattice sites with a standard deviation of 0.02 Å. We also capture anisotropic deformations of the unit cell. This was achieved by generating symmetric strain tensors, ***ϵ***, which is used to transform the lattice cell vectors as **L**_rand._ = (**I** + ***ϵ***)**L**_0_, where **L**_0_ are the original cell vectors of the simulation cell. The entries of this strain matrix are generated from the uniform distribution ${\epsilon }_{i\le j} \sim {\mathcal{U}}(-0.01,0.01)$, and internal atomic coordinates were also scaled with the cell deformation such that atoms remained at the same fractional coordinate.

To augment our database, we added disordered atomic configurations representing the liquid state. While our intention was not necessarily a thermodynamically accurate sampling of the configurational space of the liquid, we used molecular dynamics to ensure that the collected sample configurations are thermodynamically relevant. To efficiently sample the liquid phase of Ti, we utilise the (MLMD)^38^ feature of the CASTEP package. In MLMD, both DFT and MLIPs are used to calculate the PES at given points in the molecular dynamics trajectory depending on a PES calculator selection algorithm. CASTEP uses the GAP framework to achieve significant acceleration of *ab initio* molecular dynamics calculations without compromising the accuracy of the trajectories. With this framework, GAP surrogate models are generated on the fly in an automatic fashion. In particular, a MLMD simulation may be started with the first few time steps being integrated using forces obtained from DFT, while storing these labels in a database to train a GAP model. The algorithm switches between computationally expensive DFT and cheap MLIP evaluations adaptively, using DFT labels to retrain the surrogate model when necessary. Alternatively, one may also provide a training database prior to starting a MLMD simulation, where to this database is then appended further DFT evaluations in the MLMD trajectory.

The MLMD approach accelerates *ab initio* molecular dynamics as the number of time steps in a given simulation can be significantly increased, allowing the sampling of more configurations in the relevant thermodynamic phase space. We considered supercells 54 and 128 atoms for *β*-Ti, and generated volume perturbations by isotropic scaling the lattice parameter between 102 % and 90% of the ground state value. To initialise the atomic positions into a disordered state, we perform molecular dynamics using the Large-scale Atomic/Molecular Massively Parallel Simulator^39^ (LAMMPS) package with the Ti1 EAM potential by Mendelev *et al*.^40^ in the NVT ensemble. We first overheated the crystalline structure via setting the thermostat to 4000 K, followed by quenching to approximately 2000 K, and retaining atomic configurations to serve as initial geometries for MLMD. Each MLMD trajectory begins by first performing 5 initial *ab initio* steps, then a GAP model is trained and 10 further steps are computed with the surrogate model. After this, the accuracy of the surrogate model is checked against a full *ab initio* calculation, and is re-trained with the incorporation of the new *ab initio* data. The switching algorithm we utilise in MLMD is such that we adaptively change the number of steps between error checks. When the surrogate model passes the success criterion, the check interval is doubled, while the interval is halved in the case of unsuccessfully fulfilling the accuracy criterion. We consider a minimum of 10, and a maximum of 100, surrogate steps between checks. We evaluate the success of the surrogate as having less than 5 meV/atom error relative to the *ab initio* calculation.

The model complexity utilised in the GAP surrogate consisted of a 2-body kernel with 20 sparse points and cutoff distance of 4.5 Å, and a many-body SOAP descriptor with 1200 sparse points with the following descriptor hyperparameters for many body interactions: ${n}_{\max }=8$, ${l}_{\max }=8$, *r*_cutoff_ = 6.0 Å, *ζ* = 4, and *σ*_atom_ = 0.5Å. In MLMD, we utilise the Nose-Hoover thermostat with time constant of 200 fs in the NVT ensemble with a timestep of 2 fs. Across the 54 atom configurations in the MLMD trajectories a mean of 2.06 ps of simulation time was considered over 9 independent trajectories. For the 128 atom configurations, the mean simulation time was1.81 ps over 4 independent trajectories.

### Database generation for the Ti-6Al-4V alloy

For Ti-6Al-4V, we utilise and extend the NDSC method for generating bulk crystalline data^31,32^, by considering chemical perturbations on the cell similarly to vibrational BZ sampling. In this scheme we generate a series of NDSCs for each crystalline symmetry with varying levels of vibrational BZ sampling. Initially isotropic volume perturbations were considered by generating a set of NDSCs with a grid sampling of 8 × 8 × 8 for each physically observed symmetry of pure Ti. This resulted in 44, 56, and 44 starting configurations for *α*, *β* and *ω* respectively. From these, configurations containing less than 4 (*α* and *β*) and 6 (*ω*) atoms were removed as the data being constructed was concerned with targeting a dilute regime of minor alloying elements. We note that even in the largest cells we generated, the actual concentrations of the minor alloying elements vary significantly. When averaged across the database, unit cells contained 8.6 wt% Al and 5.6 wt% V, where no structures contained more than 16 wt% Al or 26 wt% V. While a very crude approximation of the target 6 wt% Al and 4 wt% V composition of the alloy, these database configurations represent a broad range of concentrations capable of capturing significant local variations of atomic environments.

Isotropic volume perturbations were generated by scaling the lattice parameters in the range of 95% to 102% of the ground state pure Ti geometry in 1% increments. Atomic positions were then perturbed around ideal lattice sites via a normal distribution with standard deviation of 0.10 Å for each volume perturbed NDSC for a total of 6 samples. The atomic species of the isotropic volume perturbed NDSCs were then randomly swapped from Ti to Al and/or V. For configurations in the *α* phase, we consider up to 1, 2 and 3 atomic species swaps from Ti to Al and/or V for configurations containing 4, 8 and 12 atoms respectively. For the *β* phase we consider up to 1 and 2 atomic species swaps for configurations of 4 and 8 atoms respectively, and, up to 1, 2 and 4 for *ω* phase configurations of 6, 12 and 24 atoms respectively. The number of atomic swaps considered is selected as a random integer on the bounds of 1 to the maximum number of swaps allowed for that configuration type. To reflect the larger proportion of Al to V in the Ti-6Al-4V, the parameters of the random sampling of chemical perturbations was adjusted to yield a mean 3:1 (Al:V) ratio across the generated strucutres. In total we generated 2064 (*α*), 2496 (*β*), and 2064 (*ω*) configurations for the isotropic volume perturbed dataset, of which 6491 were successfully evaluated with DFT.

To capture the elastic properties of Ti-6Al-4V, configurations under shear deformations were generated using NDSCs as a templates. In this instance, we utilised the following vibrational BZ sampling for each crystalline symmetry: 6 × 6 × 6 (*α*), 12 × 12 × 12 (*β*),  4 × 4 × 4 (*ω*). From these, we filter the set such that we retain only the 12 atom NDSC configurations for each crystalline system, thus resulting in 19 (*α*), 48 (*β*) and 8 (*ω*) starting geometries. Configurations with atomic positions perturbed from ideal lattice sites are then generated for each set with 16 (*α*), 6 (*β*) and 38 (*ω*) realisations for each NDSC. These configurations are then deformed via a symmetric strain tensor, scaling atomic positions, where the samples of each entry are from the uniform distribution ${\epsilon }_{i\le j} \sim {\mathcal{U}}(-0.01,0.01)$. Atomic positions were then displaced according to a normal distribution with standard deviation of 0.05 Å. Similarly, we also generate randomly deformed and chemically modified NDSCs for isotropically scaled volume perturbations. This was done by scaling the lattice parameters randomly on the interval [0.90, 1.02], for a series of copies of each NDSC with perturbed atomic positions, from which was then deformed via the strain tensor described previously. In total, targeted cell deformation data consisted of 1629 configurations, bringing the total crystalline data via this framework to 8120 configurations with 105436 atomic environments. To capture the elastic properties of Ti-6Al-4V, configurations under shear deformations were generated using NDSCs as a templates. In this instance, we utilised the following vibrational BZ sampling for each crystalline symmetry: 6 × 6 × 6 (*α*), 12 × 12 × 12 (*β*),  4 × 4 × 4 (*ω*). From these, we filter the set such that we retain only the 12 atom NDSC configurations for each crystalline system, thus resulting in 19 (*α*), 48 (*β*) and 8 (*ω*) starting geometries. Configurations with atomic positions perturbed from ideal lattice sites are then generated for each set with 16 (*α*), 6 (*β*) and 38 (*ω*) realisations for each NDSC. These configurations are then deformed via a symmetric strain tensor, scaling atomic positions, where the samples of each entry are from the uniform distribution ${\epsilon }_{i\le j} \sim {\mathcal{U}}(-0.01,0.01)$. Atomic positions were then displaced according to a normal distribution with standard deviation of 0.05 Å. Similarly, we also generate randomly deformed and chemically modified NDSCs for isotropically scaled volume perturbations. This was done by scaling the lattice parameters randomly on the interval [0.90, 1.02], for a series of copies of each NDSC with perturbed atomic positions, from which was then deformed via the strain tensor described previously. In total, targeted cell deformation data consisted of 1629 configurations, bringing the total crystalline data via this framework to 8120 configurations with 105436 atomic environments.

To gather information on the liquid phase of Ti-6Al-4V, we constructed two pure Ti supercells, 3 × 3 × 3 and 4 × 4 × 4, in the orthorhombic *β*-Ti crystalline symmetry. Utilising a multiphase pure Ti GAP developed previously, molecular dynamics in LAMMPS was preformed in the NPT ensemble on both supercells using a 2 fs timestep. The velocities of atoms were initialised from a normal distribution corresponding to a temperature of 3000 K, this was then quenched via the Nose-Hoover thermostat from 4000 K to 2500 K with a time constant of 1.35 ps over 20 ps, from which the simulation proceeded at 2500 K for an addition 20 ps to equilibrate the system. After equilibration, a series of liquidus configurations were generated by taking samples in 4 ps intervals for a total of 5 configurations for each supercell at a given pressure. We considered pressures up to 20 GPa, in 5 GPa intervals, in pure Ti when generating these configurations via the Parrinello-Rahman barostat with time constant 1.75 ps. From the 54 atom supercell samples we initialise Ti-6Al-4V configurations by randomly replacing Ti atoms with 6 Al and 2 V atoms or 13 Al and 5 V atoms in the case of the 128 atom supercell.

From these initial liquidus configurations we perform MLMD^38^ in CASTEP in the NVT ensemble. We utilise an adaptive approach to switching between *ab initio* and MLIP calculator during a single molecular dynamics simulation with identical switching criterion as we did for the liquid pure Ti, however, with the surrogate model consisting of many-body SOAP descriptors with 400 sparse points, per atomic interaction type, with the following descriptor hyperparameters: ${n}_{\max }=6$, ${l}_{\max }=8$, *r*_cutoff_ = 6.0 Å, *ζ* = 4, and *σ*_atom_ = 0.5Å. Across the 54 atom configurations in the MLMD trajectories a mean of 1.09 ps of simulation time was considered over 5 independent trajectories. For the 128 atom configurations, the mean simulation time was 0.84 ps over 16 independent trajectories.

**Fig. 1:**
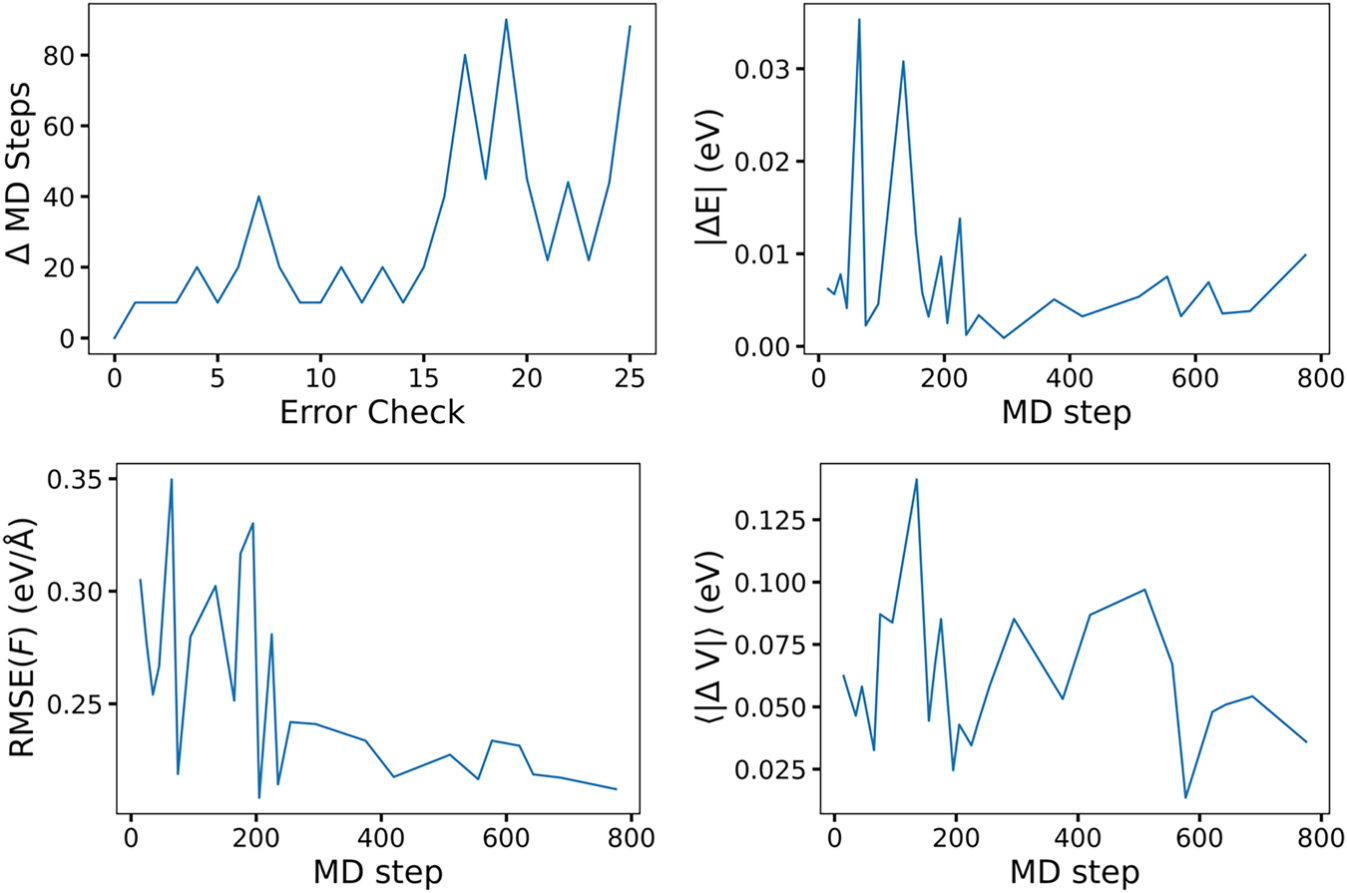
Learning rates of various quantities of interest of surrogate model for liquidus Ti-6Al-4V of during accelerated *ab initio* molecular dynamics. From left to right, top to bottom, we present the following properties: number of molecular dynamics (MD) steps between *ab initio* and surrogate calculator checks, difference between configuration energy per atom, Root Mean Squared Error (RMSE) of atomic forces, and the average absolute difference in virial stress components.

In order to assess whether enough liquid configurations were collected, we analysed the MLMD trajectories and fitted a series of GAP models. Based on our analysis, we expect that regardless of the MLIP framework used, the configurations represent an efficient sample of the liquid phase. Firstly, as an example to demonstrate the efficiency of the MLMD approach we present a series of learning curves for different quantities of interest in Fig. 1. In this MLMD trajectory we were simulating 128 atoms of Ti-6Al-4V at 2500 K starting from a configuration corresponding to 0 GPa in pure Ti. We observe that most of the gains in the accuracy of the surrogate model are achieved from configurations gathered in first quarter of a given MLMD trajectory over 1.6 ps.

**Fig. 2:**
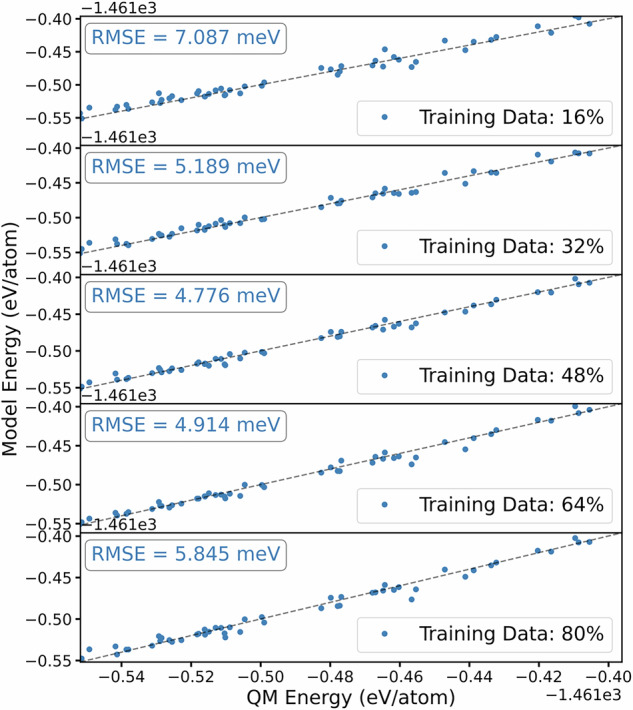
Model performance on configurational energy for Ti-6Al-4V GAP models developed with increasing liquidus data against a validation set.

During the data collection process, we also evaluated the rate of learning of a surrogate across multiple trajectories. We firstly concatenated all liquidus data, from which we partitioned 20% as an evaluation set and 80% as a training dataset. From the 80% partition, we train a series GAP models for increasing quantity of data as illustrated in Fig. 2 and evaluate the RMSE on training observables. We note from Fig. 2 that the improvement with increasing amount of data is non-monotonic such that models with greater than 48% of available liquidus data no longer provided additional benefit, at least using the same GAP model hyperparameters. The total size of the liquidus Ti-6Al-4V dataset consisted of 304 configurations with 38912 atomic environments.

## Data records

We make available the databases and presented models in a dedicated Zenodo repository^41^.

### File format

The atomic structures, containing the DFT total energy (eV), force (eV/Å) and virial stress (eV) labels are stored in the extended XYZ format (https://github.com/libAtoms/extxyz). Standard tools, such as the Atomic Simulation Environment^42^ may be used to parse and convert the files. The sample GAP models are provided as XML files, which can be used via the quippy package (https://github.com/libAtoms/quip). The fitted ACE models are provided both in the JSON and YACE file formats, loadable via the package ACEPotentials.jl (https://github.com/ACEsuit/ACEpotentials.jl) and LAMMPS^39^, respectively.

## Technical Validation

Databases intended to be used to fit MLIP are typically synthetic: structural models are generated algorithmically to provide example atomic environments which are intended to represent thermodynamically relevant regions of the PES. Due to the high dimensionality of the PES, such databases are inevitably incomplete as it is neither possible nor practical to generate atomic structures that uniformly cover the 3*N* dimensional configuration space. Therefore, the utility of a database of atomic configurations should be demonstrated, ideally, by fitting MLIP.

In order to validate the generated database, we have fitted a series of MLIP models, using the GAP and ACE frameworks, and evaluated these models on our benchmark data set. Naturally, the database is not limited to either of these frameworks, and indeed, models with other schemes or more careful hyperparameter tuning may easily outperform the benchmarks presented here. Our intention is to demonstrate the coverage of the training database and showcase our benchmarks which are intended to be a stringent test of any surrogate model, covering a broad range of thermodynamically relevant conditions.

### Multiphase Ti

#### Fitting Machine Learned interatomic Potentials

The GAP model developed on the multiphase Ti dataset is constructed using a 2-body inverse polynomial kernel alongside the SOAPTurbo kernel. The GAP model consisted of a total 3320 sparse points, 3300 of which are contained in the SOAPTurbo kernel. The representative atomic environments for the SOAPTurbo kernel were selected from CUR decompositions^43^ of each configuration type within the data set, ensuring a broad coverage of points across each configuration type. In the case of the 2-body kernel, points were selected uniformly by taking a representative environment from each bin of a histogram of evaluations with the 2-body kernel, described as uniform in Klawohn *et al*.^44^. The 2-body kernel is short range, acting within 3.5 Å with an inverse polynomial basis with exponents -4, -8, -12 and -14, primarily ensuring that the GAP model is repulsive at short atomic distances to stop non-physical atomic overlaps such that MD simulations remain stable. The SOAPTurbo kernel serves to capture the many body interactions within the dataset. We utilise the SOAPTurbo kernel hyperparameters^45^): basis complexity of ${n}_{\max }$=8 and ${l}_{\max }$= 8 with cut off distances of *r*_soft_ = 5.5 Å, *r*_hard_ = 6.0 Å, atomic-centred Gaussian widths *σ*_∥_ = *σ*_⊥_ = 0.5 Å, and kernel exponent *ζ* = 6. The reader is referred to Klawohn *et al*.^44^ for further details on the functionality and parameters within of GAP and descriptors as implemented in QUIP.

Alongside the GAP, an Atomic Cluster Expansion (ACE)^24^ is also presented as a computationally lightweight alternative model, implemented via the ACEpotentials.jl^46^ suite. Within ACEpotentials.jl, the maximum complexity is collapsed to a single number called the *total degree* which can be set independently for each correlation order *ν*. The last step in building an ACE potential is using a regression framework to fit coefficients which map input coordinates to observed quantities, in our case DFT labels. Of the multiple choices for regression frameworks implemented within ACEpotentials.jl, and the Bayesian linear regression optimiser was utilised in our work. A series of ACE potentials were fit the reproduce the DFT labels for combinations of *ν* and *total degree*, with *ν* ranging from 3 to 5, and *total degree* from 16 to 20. We observed that utilising *ν* ≥ 4 resulted in significant over-fitting of the multiphase Ti dataset, and increasing the *total degree* monotonically reduced the training RMSE within *ν* = 3. A spatial cut-off of 6 Å was utilised for all ACE models considered. In the final version used *ν* = 3, for a *total degree* of 20 across each correlation order, resulting in a total of 1809 basis functions.

**Fig. 3:**
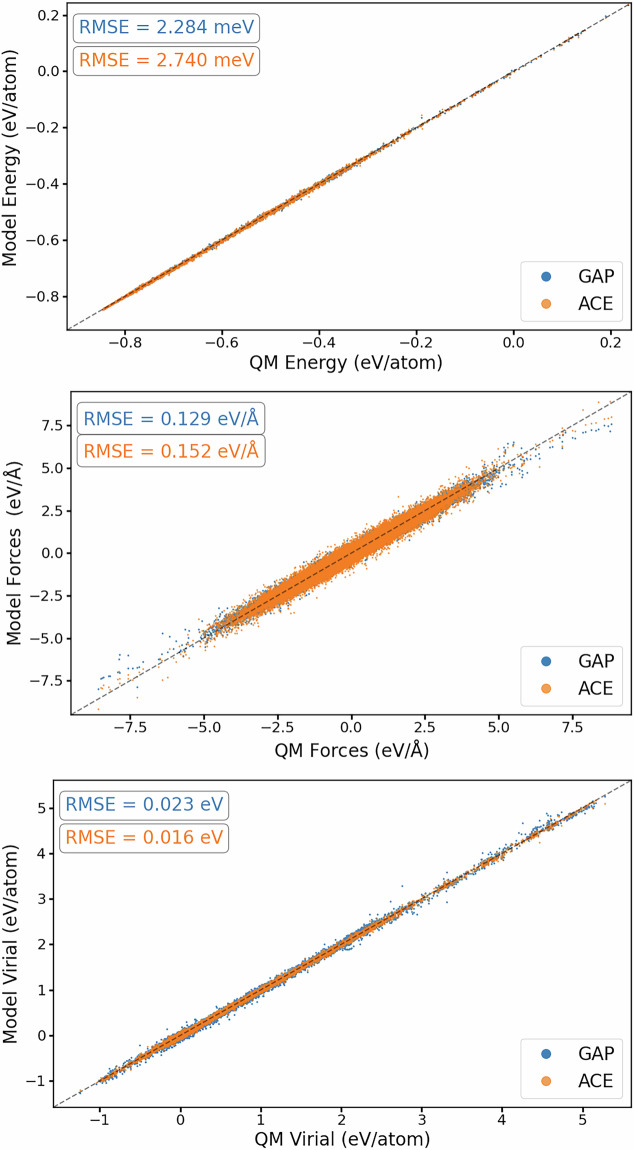
Performance of surrogate models against training observables of the training dataset for each model trained on the multiphase Ti dataset. The top, middle and bottom panels show the correlations of predicted and target energies, force and virial stress components, respectively. For plotting, energy values have been shifted.

After generating the *ab initio* database through the strategies outlined above, a series of GAP and ACE models were trained until the final set of hyperparameters for each model was realised. The surrogate models presented here are trained on the total configurational energy, atomic forces and virial stresses, and we evaluate the model performance on reproducing these quantities in Fig. 3. From the figure, we compare each surrogate model’s prediction with that of the *ab initio* calculation and compute the RMSE for each quantity using the entire database. This constituted 6640 DFT observations, for a total of 82096 atomic environments. As indicated by Fig. 3, both the GAP and ACE potential accurately reproduce the underlying data with uncertainty being on the order of meV per atom.

#### Elastic Constants

We have calculated the lattice parameters and elastic constants of the crystalline phases of Ti using our GAP and ACE models and compare them to DFT benchmark values, presentedin Tables 1, 2 and 3 for *α*-Ti, *ω*-Ti and *β*-Ti respectively. We find that both surrogate models can reproduce the ground state ambient pressure geometry associated with each crystalline symmetry considered. As is in agreement with other theoretical investigations in literature^7,10,11,47,48^ the *ab initio* calculations performed in this work find that *ω*-Ti is the ground state geometry at ambient pressure. Computed using DFT, the energy differences of the phase transitions *α* → *ω* and *α* → *β* are *ΔE*_*α*→*ω*_ = − 5.4 meV/atom and *ΔE*_*α*→*β*_ = 111.9 meV/atom, respectively. Both the GAP and ACE potentials fitted by us reproduce the energy difference between these crystalline phases as: $\Delta {E}_{\alpha \to \omega }^{{\rm{GAP}}}=-\,5.4$ meV/atom and $\Delta {E}_{\alpha \to \beta }^{{\rm{GAP}}}=-\,110.6$ meV/atom, and $\Delta {E}_{\alpha \to \omega }^{{\rm{ACE}}}=-\,5.5$ meV/atom and $\Delta {E}_{\alpha \to \beta }^{{\rm{ACE}}}=109.6$ meV/atom. In addition, we find that the ground state lattice parameters predicted for each system is in good agreement with previous *ab initio* studies at the same level of theory by Mei *et al*.^11^, Hu *et al*.^47^ and Nitol *et al*.^48^. The surrogate models also reproduce the elastic properties across the different crystalline symmetries, with particularly good agreement for *ω*-Ti with a (MAE) across the all elastic constants of 1.5 GPa (1.7%) and 1.0 GPa (0.9%) for GAP and ACE respectively. The elastic properties of the DFT calculations performed here are also in good agreement with value reported in previous literature^47,48^.

**Table 1:** Elastic constants and lattice cell parameters for *α*-Ti.

*α*-Ti	DFT	GAP	ACE
Elastic constants (GPa):			
*C*_11_	180.2	173.7	175.5
*C*_33_	189.1	185.0	193.3
*C*_12_	79.0	83.5	86.7
*C*_13_	76.3	79.4	73.9
*C*_44_	44.6	37.4	43.8
*C*_66_	50.6	45.1	44.4
*B*	112.5	113.0	112.6
Lattice parameters:			
*a* (Å)	2.939	2.938	2.938
*c* (Å)	4.647	4.648	4.648
*V*_0_ (Å^3^/atom)	17.38	17.38	17.38

**Table 2:** Elastic constants and lattice cell parameters for *ω*-Ti.

*ω*-Ti	DFT	GAP	ACE
Elastic constants (GPa):			
*C*_11_	195.0	195.0	194.6
*C*_33_	247.5	244.3	243.6
*C*_12_	81.1	84.5	80.3
*C*_13_	52.7	51.6	53.8
*C*_44_	54.4	53.3	54.9
*C*_66_	57.0	55.3	57.1
*B*	112.0	112.1	111.8
Lattice parameters :			
*a* (Å)	4.579	4.579	4.579
*c* (Å)	2.831	2.830	2.830
*V*_0_ (Å^3^/atom)	17.13	17.13	17.13

**Table 3:** Elastic constants and lattice cell parameters for *β*-Ti.

*β*-Ti	DFT	GAP	ACE
Elastic constants (GPa):			
*C*_11_	91.0	87.9	86.5
*C*_12_	112.4	114.5	117.0
*C*_44_	40.3	45.5	39.6
*B*	105.2	105.6	106.8
Lattice parameters:			
*a* (Å)	3.254	3.254	3.254
*V*_0_ (Å^3^/atom)	17.23	17.23	17.23

**Fig. 4:**
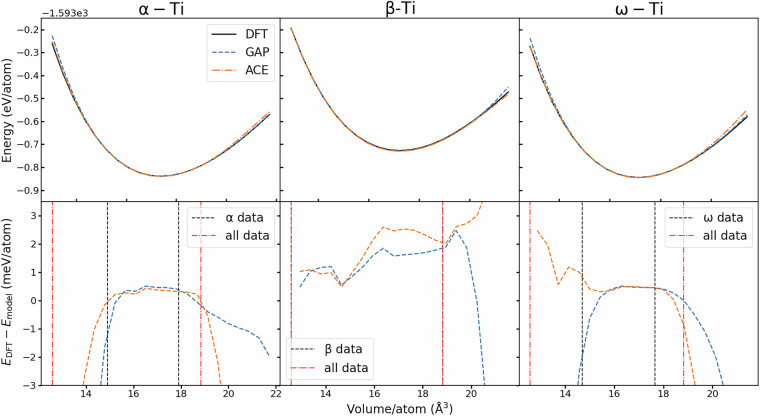
Energy-volume curves (top) and the difference between the surrogate models and underlying DFT (bottom) for each crystal symmetry of Ti. Dashed red lines indicate the bounds where there exists associated crystalline data in the model training, and black dashed indicate that specific to the crystal symmetry.

We also characterise the variation of potential energy variation due to volume perturbations, presented in Fig. 4, showing that both the GAP and ACE potentials accurately capture the bulk modulus of each crystalline system. To demonstrate how the accuracy of the surrogate models depend on the coverage of the training data, we plotted the difference between the predicted energy compared to the DFT data, also indicating the range the atomic volumes present in the training set of configurations.

Our results indicate that the *α*, *β* and *ω* phases of Ti are well represented in the database around the equilibrium lattice constants. The range includes higher volumes, which correspond to expanded cells at elevated temperatures as well as lower volumes corresponding to moderately high pressures. As no high-pressure phases are included in the training set, it is not expected that complex phase transitions at high pressures would be captured by an MLIP fitted using our data.

These energy-volume curves demonstrate that the surrogate models are accurate where there exists the respective training data, as denoted by the hashed boundaries within Fig. 4 for a given crystalline symmetry within the crystalline subset of the whole database. We also observe that for the crystalline phases and immediately around the ground state volume, the energy predicted by both surrogates is somewhat less than that of the reference DFT calculation. In these tests, if an atomic configuration has a specific volume outside of the range represented by the training data, the GAP model always predicts the potential energy to be greater than the reference DFT in all instances of extrapolation, whereas, the ACE model shows in low-volume *ω*-Ti and high-volume *β*-Ti a lower potential energy than the reference.

#### Vibrational Properties

Another common benchmark within MLIP development is how well the Force Constant Matrix (FCM) is reproduced, as this assesses the curvature of the PES with respect to atomic displacements. How well a model reproduces the FCM can be represented by presenting the phonon dispersions and the corresponding density of states for each crystalline symmetry of interest, providing quantitative insight into how well a model predicts forces in the harmonic regime of the PES. In our study, the FCMs were calculated for the developed MLIPs using the finite difference method^49^ as implemented in the phonopy package^50^. We consider the dispersion and density of states for the minima in the PES of bulk Ti as represented by *α*-Ti and *ω*-Ti, and also at the saddle point represented by the dynamically unstable *β*-Ti phase.

**Fig. 5:**
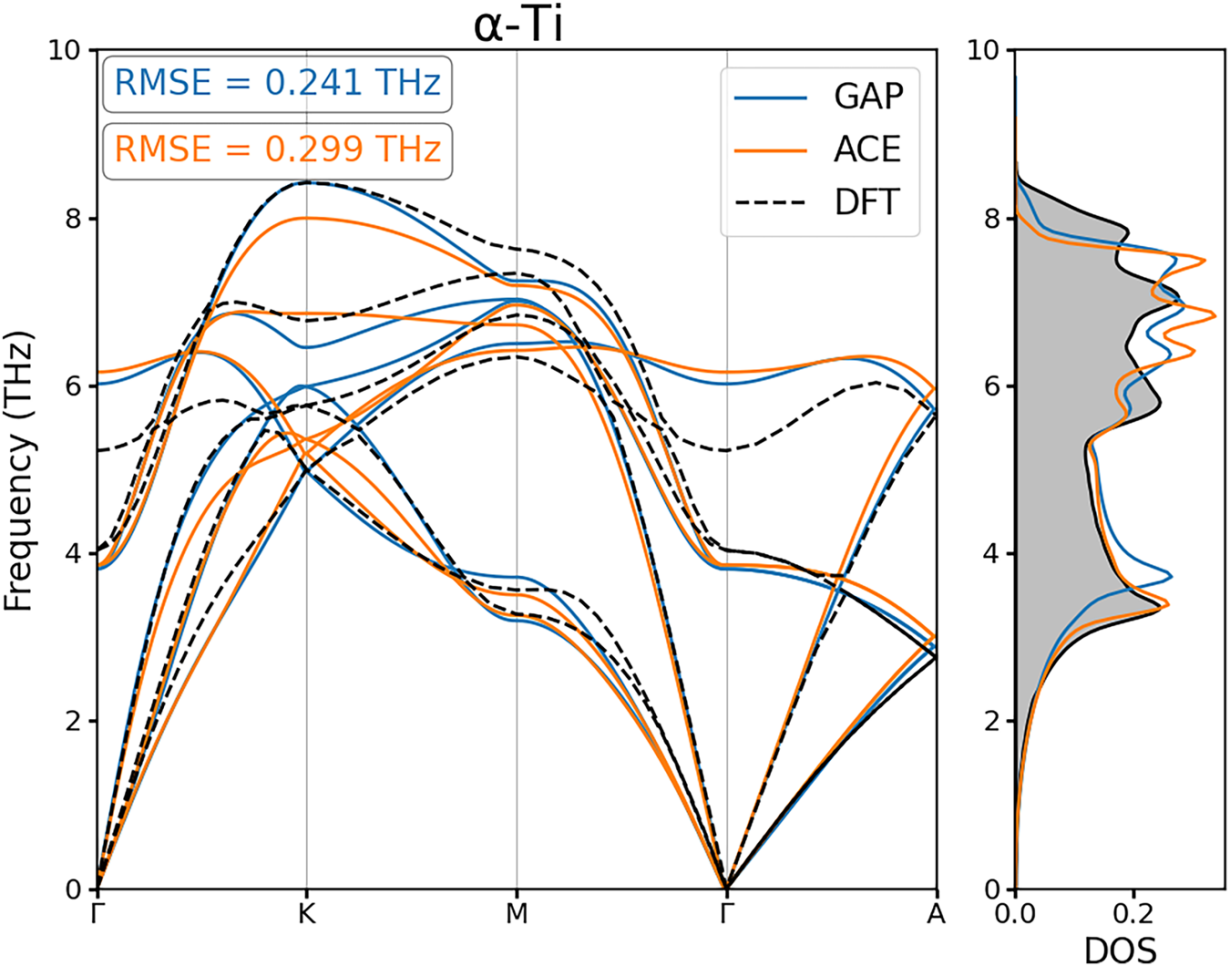
Phonon dispersion and density of states for *α*-Ti as calculated by the reference DFT calculation alongside GAP and ACE surrogate models developed.

**Fig. 6:**
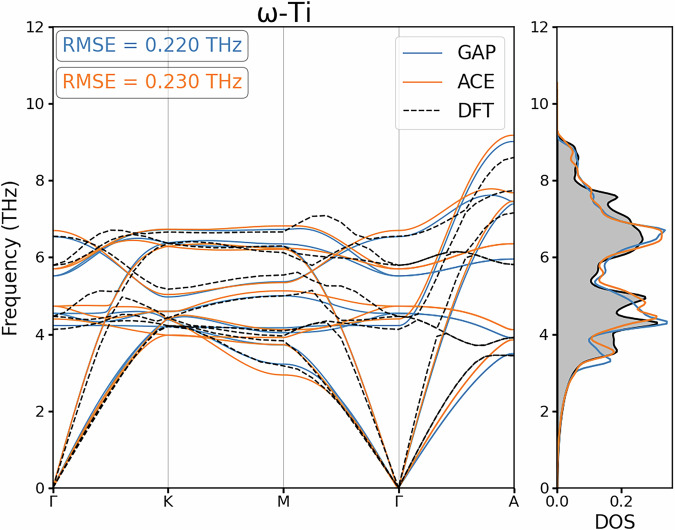
Phonon dispersion and density of states for *ω*-Ti as calculated by the reference DFT calculation alongside GAP and ACE surrogate models developed.

**Fig. 7:**
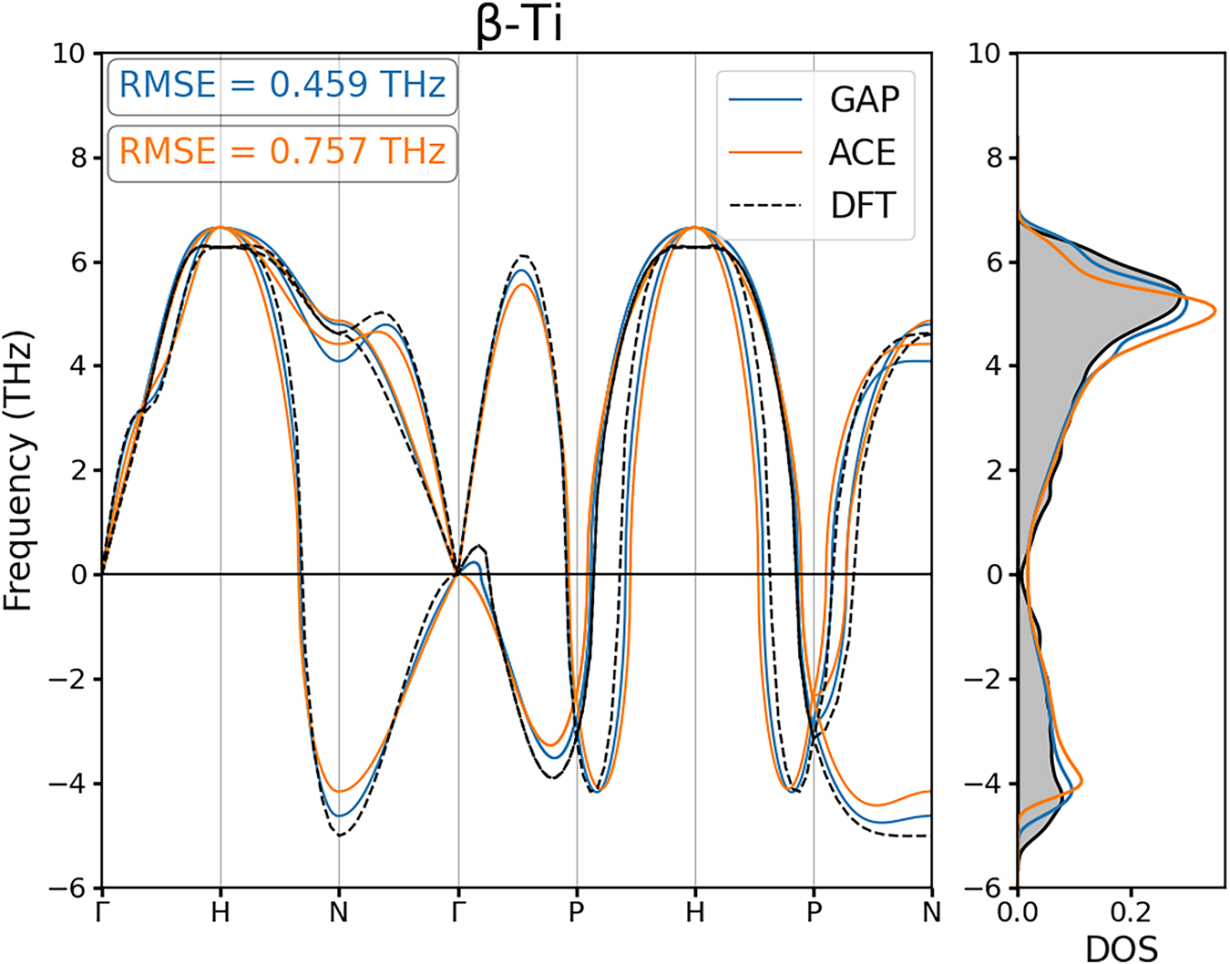
Phonon dispersion and density of states for *β*-Ti as calculated by the reference DFT calculation alongside GAP and ACE surrogate models developed.

The phonon dispersions are shown along high-symmetry lines within the vibrational BZ^51^ for our underlying *ab initio* calculator and surrogate models in Figs. 5, 6 and 7 respectively. For all crystalline phases, we found excellent agreement between the GAP and ACE potential with our DFT calculations, and this was achieved in an efficient manner, by using data that specifically targets the vibrational properties of each crystalline phase via the NDSC method within our database building. Our phonon dispersions are in excellent agreement with Hu *et al*.^47^, whilst some qualitative disagreement appears compared to the results presented by Nitol *et al*.^48^ in case of *α*-Ti and *β*-Ti.

### Ti-6Al-4V

#### Fitting Machine Learned interatomic Potentials

We developed a series of multiphase potentials for the Ti-6Al-4V alloy. A careful study of hyperparameters resulted in the final iteration of the GAP model, which was constructed utilising 2-body kernels and many-body terms using the SOAPTurbo descriptor. The 2-body kernels utilised an inverse polynomial basis set with exponents −4, −8, −12 and −14 with spatial cutoff of 3.5 Å, where 20 uniformly selected sparse points were used per elemental interaction type. The SOAPTurbo kernels consisted of 3563, 3700, and 2492 sparse points for Al, Ti and V centred environments, respectively. The following SOAPTurbo hyperparameters are utilised in the final iteration of our GAP surrogate: ${n}_{\max }$ = 8, ${l}_{\max }$ = 8, *r*_soft_ = 5.5 Å, *r*_hard_ = 6.0 Å, *σ*_∥_ = *σ*_⊥_ = 0.5 Å, and *ζ* = 6.

The database of atomic configurations were generated using the procedure we outlined earlier, subsequently used to fit a GAP surrogate model which was able to generate stable MD trajectories across a broad range of thermodynamic conditions. However, when the same database was used to fit an ACE model, we observed that the V-V interaction was poorly characterised such that V mobility in molecular dynamics simulations was too great and non-physical interatomic distances were recorded. To address this problem, we included additional configurations to capture V-V interactions. In the configurations representative of Ti-6Al-4V via the stoichiometries *α* − Ti_42_Al_5_V_7_, *β* − Ti_19_Al_3_V_5_, and *ω* − Ti_26_Al_4_V_6_, we fixed the positions of a pair of V-V nearest neighbours, and performed a geometry optimisation with DFT relaxing all the other atoms. We then displaced one of the V atoms such that the its distance to its nearest V neighbour was less than 1.6 Å. We also performed active learning, which included a set of configurations obtained from performing molecular dynamics on each crystal symmetry containing the stoichiometry Ti_8_Al_1_V_2_, taking snapshots at 0.5 ps intervals, using the corresponding supercells: 2 × 2 × 3 (*α*), 3 × 3 × 3 (*β*), and 2 × 2 × 3 (*ω*). The total size of the final iteration of the dataset was 8507 configurations with 147522 atomic environments. The final version of the ACE potentials presented here utilised *ν* = 3, for a *total degree* of 15 across each correlation order, totalling 29,703 basis functions. To keep our developed MLIP commensurate, we also included this additional data in our final version of our GAP surrogate that was not described earlier.

Alongside all of our ACE models we also considered a second variant of our ACE model, labelled ACE2, which includes additional data designed to capture the atomic environments in our benchmark tests. We intended to demonstrate whether the errors observed in our benchmark tests are due to insufficient coverage of the training set or a limitation of the functional form specified by our MLIP hyperparameters. To train ACE2, we generated NDSCs commensurate with a phonon grid sampling of 2 × 2 × 2 for each phonon benchmark configuration, where atomic positions were displaced according to a normal distribution of standard deviation 0.05 Å, to generate a total of 6 examples for every NDSC of each phonon benchmark configuration. This additional data constituted 374 DFT calculations with 6500 atomic environments. A new ACE model was trained, appending the targeted data to the original dataset, with the weights on these targeted observables set as: ***w***_*E*_ = 30, ***w***_*F*_ = 25, and ***w***_*V*_ = 5. The ACE2 model may be interpreted as a best possible improvement at a given surrogate model hyperparameter set, and serves as a metric to understand the quality of the original ACE which had no data that explicitly targeted these types of benchmarks.

**Fig. 8:**
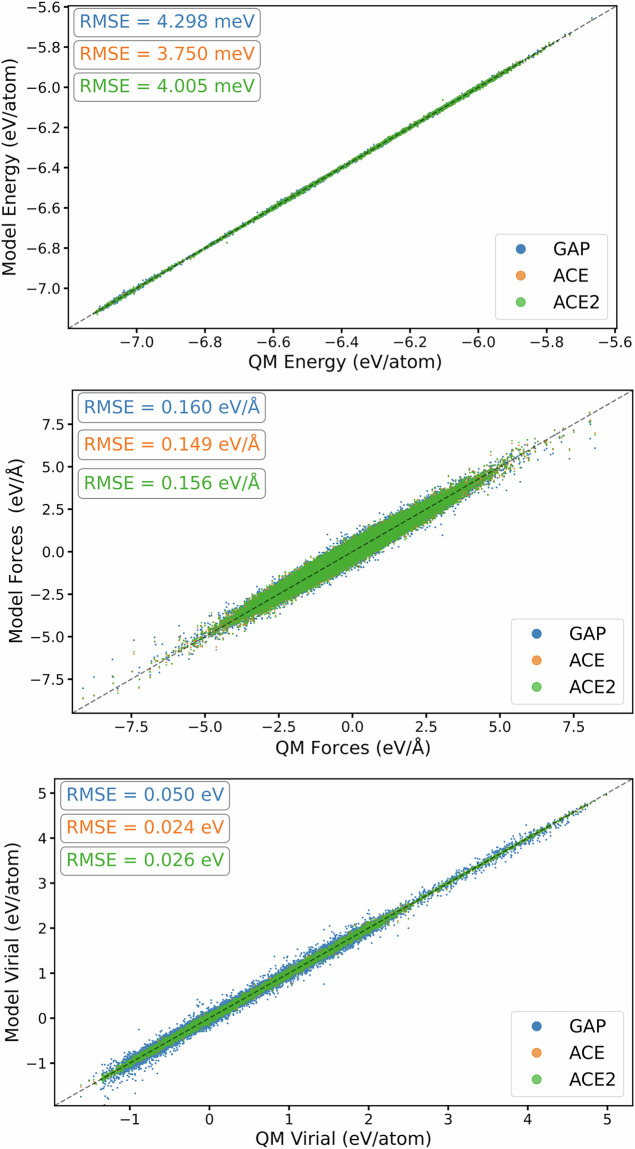
Performance of surrogate models compared to training observables for each model in Ti-6Al-4V. The top, middle and bottom panels show the correlations of predicted and target energies, force and virial stress components, respectively. For plotting, energy values have been shifted.

The MLIP developed in this work are trained on the total configurational energy, atomic forces and virial stresses, and we evaluate the model performance of the final iteration of models developed on reproducing these quantities in the total training data in Fig. 8. We compare each surrogate model’s prediction with the DFT prediction and compute the RMSE for each quantity using the full dataset, which constituted 8507 *ab initio* calculations for a total of 502115 observables. As indicated by the figure, both GAP and ACE surrogates reproduce the underpinning training data energy labels with accuracy of the order of meV/atom. Compared to training results presented for our multiphase Ti potential in Chapter, we note that the RMSE of our multiphase Ti-6Al-4V surrogate models are comparable to the single element multiphase Ti models, despite the added complexity arising from chemical permutations.

**Fig. 9:**
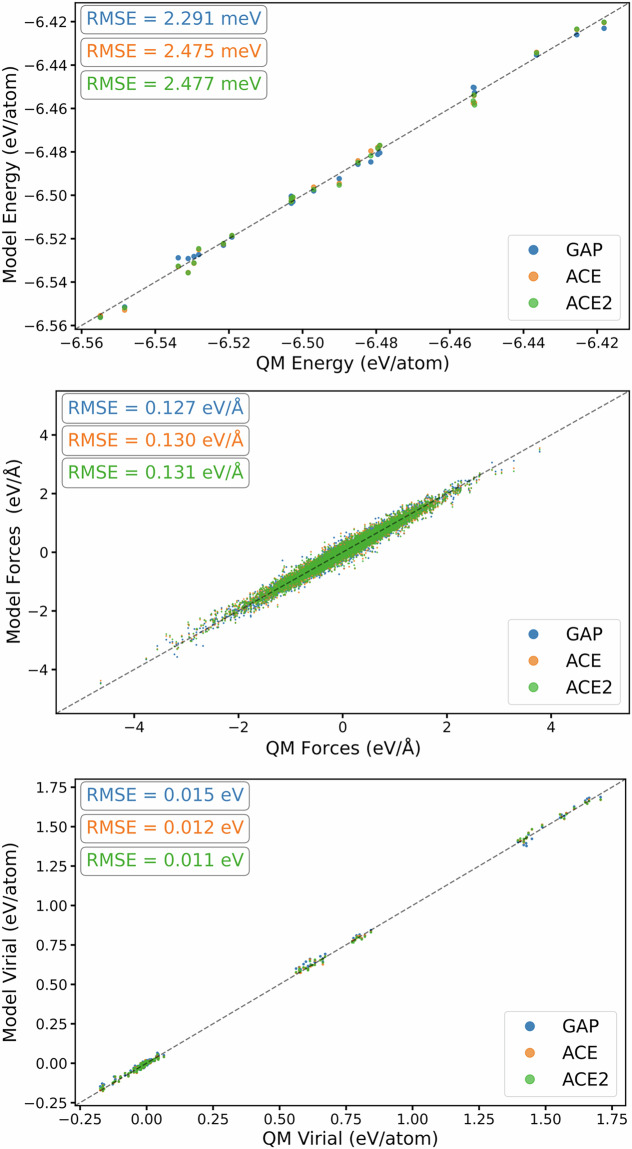
Performance of surrogate models compared to observables in a validation datasetfor each model in Ti-6Al-4V. The top, middle and bottom panels show the correlations of predicted and target energies, force and virial stress components, respectively. For plotting, energy values have been shifted.

To test the ability of the NDSC approach to gather relevant configurations in a multi-elemental bulk phase, we evaluate our models against a validation dataset of configurations that represent the Ti-6Al-4V stoichiometry, where the details of its construction as discussed earlier. The total validation dataset constitutes 23 configurations with 2604 atomic environments and 7973 observables. In Fig. 9, we present the performance of both surrogate models by comparing the predicted configurational energy, atomic forces and virial stresses in this validation set, and find that both models reproduce the underlying *ab initio* calculations to similar accuracy of that of the training observables.

#### Elastic Constants

We computed the elastic properties of the cells representative of the Ti-6Al-4V stoichiometry. In these benchmarks, as described earlier, we utilise examples of each crystalline symmetry of large supercells containing 54, 64, and 81 (*α*, *β* and *ω*, respectively) atoms. When computing the elastic constants using finite differences, prior to the deforming the lattice, geometry relaxation using the appropriate MLIP model was carried out to provide the ground state lattice parameters and atomic positions. When calculating macroscopic elasticity properties using numerical differentiation, atomic positions of each finite strain configuration should be relaxed. However, this approach would have incurred a significant additional computational cost when calculating the reference values using DFT, due to the large number of atoms in the configurations. As the main purpose of this calculation is to provide benchmark values, to be used in validating MLIP models, we decided to keep the atomic positions unrelaxed. As a result, our reported elastic constants are not commensurate with experimental observations and therefore not relevant in characterising the macroscopic properties of Ti-6Al-4V, only serve to provide insight on the quality of interpolation by the MLIP developed in this work. We refer to these elastic constants as *unrelaxed*, which are presented in Tables 4, 5 and 6.

**Table 4:** Unrelaxed elastic constants and lattice parameters for *α*-Ti-6Al-4V at 0 K utilising a 3 × 3 × 3 supercell.

*α*-Ti-6Al-4V 0 K	DFT	GAP	ACE	ACE2
Elastic constants (GPa):				
*C*_11_	198.3	197.7	199.4	199.65
*C*_33_	198.2	221.3	208.4	209.2
*C*_12_	67.2	69.2	65.4	66.6
*C*_13_	73.8	61.0	70.5	68.1
*C*_44_	48.9	41.4	49.6	49.0
*C*_66_	65.6	64.2	67.0	66.5
*B*	113.8	111.0	113.2	112.6
Lattice parameters:				
*a* (Å)	8.739	8.739	8.737	8.736
*c* (Å)	13.887	13.888	13.885	13.882
*V*_0_ (Å^3^/atom)	17.01	17.01	17.00	16.99

**Table 5:** Unrelaxed elastic constants and lattice parameters for *β*-Ti-6Al-4V at 0 K utilising a 4 × 4 × 4 supercell.

*β*-Ti-6Al-4V 0 K	DFT	GAP	ACE	ACE2
Elastic constants (GPa):				
*C*_11_	153.0	160.2	154.2	156.5
*C*_12_	90.7	81.6	84.1	84.4
*C*_44_	60.9	74.2	61.2	63.1
*B*	111.5	107.8	107.5	108.5
Lattice parameters:				
*a* (Å)	11.231	11.231	11.237	11.240
*V*_0_ (Å^3^/atom)	17.04	17.04	17.07	17.08

**Table 6:** Unrelaxed elastic constants and lattice parameters for *ω*-Ti-6Al-4V at 0 K utilising a 3 × 3 × 3 supercell.

*ω*-Ti-6Al-4V 0 K	DFT	GAP	ACE	ACE2
Elastic constants (GPa):				
*C*_11_	195.9	198.5	190.7	190.8
*C*_33_	229.1	254.4	215.6	218.5
*C*_12_	84.5	91.5	84.1	83.1
*C*_13_	57.1	43.3	56.3	55.8
*C*_44_	51.0	46.5	51.3	50.6
*C*_66_	55.7	53.5	53.3	53.9
*B*	113.1	111.9	110.1	110.0
Lattice parameters:				
*a* (Å)	13.670	13.678	13.673	13.678
*c* (Å)	8.465	8.469	8.466	8.469
*V*_0_ (Å^3^/atom)	16.91	16.94	16.92	16.94

Using a fast surrogate model allows us to predict elastic constants at finite temperatures, which can be related to experimental observables. We used our ACE surrogate model to run MD simulations in the canonical ensemble and computed the elastic constants from the fluctuation of the stress tensor elements^52–54^. We initialised a series of supercells with the orthorhombic versions of the *α*-Ti symmetry with 13 × 8 × 9 repeating units, randomly replacing Ti with Al and V to construct the Ti-6Al-4V stoichiometry. The LAMMPS package was used to propagate the dynamics and to monitor stress fluctuations for different strain patterns via the computation of the Born matrix. We compare our results to single crystal^55^ and polycrystalline^56^ experiments at room temperature alongside a theoretical result at the Generalized Gradient Approximation (GGA) DFT level of theory^57^ in Table 7.

**Table 7:** Finite temperature elastic constants and cell parameters for *α*-Ti-6Al-4V at 300 K compared to literature values.

*α*-Ti-6Al-4V	ACE	Ex_1_^55^	Ex_2_^56^	DFT^57^
300 K				
E.C. (GPa):				
*C*_11_	158.5 (3.1)	168.0	143.0	153.2
*C*_12_	89.8 (3.3)	108.0	110.0	55.1
*C*_13_	71.4 (0.7)	39.0	90.0	48.6
*C*_33_	189.2 (1.0)	144.0	177.0	157.2
*C*_44_	43.3 (0.5)	44.0	40.0	37.1

The column Ex_1_ corresponds to a single crystal experiment^55^, whereas Ex_2_ shows elastic constants determined on polycrystalline samples^56^. Predictions using DFT are also presented^57^.

Our elastic constants, as predicted by our surrogate ACE model, are found to be consistently between the single and polycrystalline experimental results, however, our model tends to over-predict the stiffness with respect to strains in the *ϵ*_33_ direction. We also provide uncertainty estimates on our results that arise from different local permutations of the minor alloying components, and observe that the local ordering in a single crystal has a negligible effect on elastic constants. At 0 K, we observed similar results in *α*-Ti-6Al-4V with the finite differences method, where we computed 20 different realisations for supercells used previously to benchmark our MLIP against our unrelaxed reference DFT elastic constants, however, this time allowing for internal relaxation of atomic positions. We similarly computed the 0 K *β*-Ti-6Al-4V elastic constants for many random permutations, however, noting that due to the dynamic instability of the *β* phase, we do not relax atomic positions, and find that local ordering contributes variability less than 5 GPato the elastic constants in our ACE model.

#### Vibrational Properties

To evaluate how accurately the FCM are reproduced by the MLIP developed on the Ti-6Al-4V dataset, we compute phonon dispersions and density of states for a series of configurations that are tractable to high-symmetry points beyond Γ-point predictions with our *ab initio* reference method. The high computational cost of DFT calculations puts a limit on the size of our unit cells, and as a consequence, the concentration of the minor alloying components Al and V are consequently higher than in the Ti-6Al-4V alloy. We considered configurations where the minor alloying components were nearest neighbours in the crystalline lattice for eachsymmetry. We note that Al-Al, Al-V and V-V interactions are only sparsely represented in the training database, therefore our benchmarks can be regarded as a stringent tests of extrapolative behaviour of the models.

**Fig. 10:**
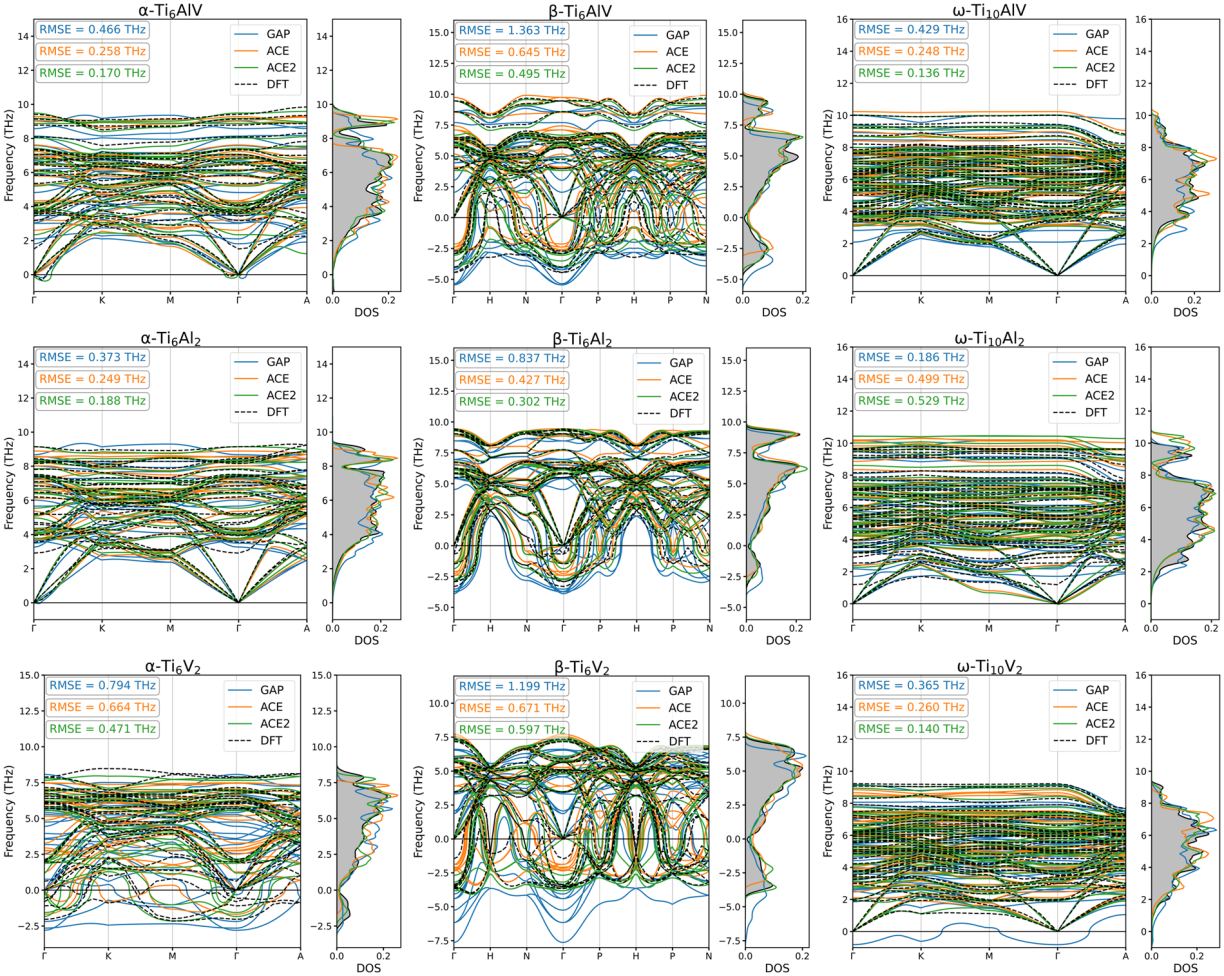
Phonon dispersion and density of states as calculated with reference DFT and developed MLIP on the Ti-6Al-4V dataset. Minor alloying components (Al and V) are placed on nearest neighbouring sites.

We computed the FCM as predicted by MLIP with the finite difference method using phonopy and from the FCM we determine the phonon dispersion and density of state relations. We show the phonon dispersions and density of states in Fig. 10. Despite no training data point was specifically crafted to represent these configurations, we observed generally good agreement in most cases between the DFT reference and our MLIP models. However, discrepancies in case of some of the phonon dispersion benchmark tests remain present. In order to quantify whether inaccuracies in the surrogate models are due to insufficient training data or inadequate choice hyperparameters, such as the spatial cutoff distance or the body order representation, we carried out further numerical experiments.

We hypothesised that augmenting the original training data with data points specifically representing configurations that contain Al-Al, Al-V and V-V atom pairs at nearest neighbour crystalline sites would improve the accuracy of the predicted vibrational properties, if the reason for inaccuracies are due to inadequate data coverage.

To gain more insight into the relevance of our original dataset in representing atomic environments in our benchmark unit cells, we also fitted another MLIP, labelled by ACE2, as described in. The phonon dispersion RMSE across all systems with the ACE2 model, based on data targeted to represent our benchmarks, shows an average improvement of 21%, which is similar to the improvement seen across phonon dispersion relations of ACE over the GAP model.

To provide further confirmation to our hypothesis, we analysed the similarity of atomic environments found in the original and extended datasets, and comparing them to the atomic environments found in the configurations used for the phonon benchmark study. We calculated the similarity, or covariance values, *C*_*ij*_ of two atomic environment *i* and and *j* using their SOAP descriptors **v**_*i*_ and **v**_*j*_ as

$${C}_{ij}={({{\bf{v}}}_{i}\cdot {{\bf{v}}}_{j})}^{\zeta }$$

using *ζ* = 4. Using this measure of similarity, *C*_*ij*_ = 1 corresponds to identical atomic environments and lower values signify different atomic environments.

**Fig. 11:**
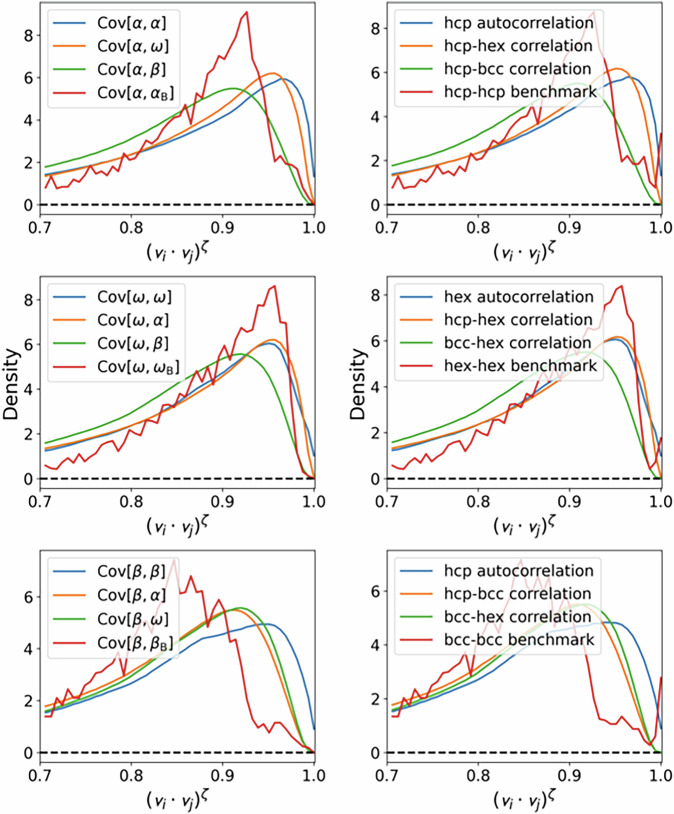
Histogram of SOAP similarity values. The left column represents the original database and the right column represents the database augmented with targeted data points. The three rows represent subsets of the training databases containing *α*, *β* and *ω* phase configurations compared to atomic configurations in each phase (blue, orange and green lines) as well as the unit cells used in the phonon benchmarks (red lines). Blue lines represent the similarities of atomic environments belonging to the same phase.

All pairwise similarity values were calculated, and their histograms are presented in Fig. 11. We grouped the values such that we present the similarities of environments found in the configurations used for the phonon benchmarks and those in the training databases representing the *α*, *β* and *ω* phases. The histograms emphasise that atomic environments in the *α* and *ω* phases are more similar to each other than to those in the *β* phase. It is also revealed that in the original database the atomic environments typical for the phonon benchmark configurations are under-represented, which is remedied when augmenting the dataset using further NDSC data points.

Given that our phonon benchmark tests are only indicative of the extrapolative behaviour of the generated MLIP models, we conclude that the Ti-6Al-4V dataset is sufficient to produce MLIP that accurately characterise the vibrational properties of the Ti-6Al-4V alloy where the concentrations of the minority components closely reflect those of the real material.

## Data Availability

DFT and MLMD calculations were performed using version 24.1 of the CASTEP code, which is available freely for academic users under an academic licence (https://licences.stfc.ac.uk/product/castep), or through the Materials Studio software package (https://www.3ds.com/products/biovia/materials-studio). NDSCs were generated using Python code available under the repository https://github.com/ConnorSA/ndsc_tut.
